# BAP1-defficient breast cancer in a patient with BAP1 cancer syndrome

**DOI:** 10.1007/s12282-022-01354-0

**Published:** 2022-04-05

**Authors:** Ana Blatnik, Domen Ribnikar, Vita Šetrajčič Dragoš, Srdjan Novaković, Vida Stegel, Biljana Grčar Kuzmanov, Nina Boc, Barbara Perić, Petra Škerl, Gašper Klančar, Mateja Krajc

**Affiliations:** 1 Department of Clinical Cancer Genetics, Institute of Oncology Ljubljana, Zaloška cesta 2, 1000 Ljubljana, Slovenia; 2 Division of Medical Oncology, Institute of Oncology Ljubljana, Ljubljana, Slovenia; 3 Department of Molecular Diagnostics, Institute of Oncology Ljubljana, Ljubljana, Slovenia; 4 Department of Pathology, Institute of Oncology Ljubljana, Ljubljana, Slovenia; 5 Department of Radiology, Institute of Oncology Ljubljana, Ljubljana, Slovenia; 6 Division of Surgery, Institute of Oncology Ljubljana, Ljubljana, Slovenia; 7 Biotechnical Faculty, University of Ljubljana, Ljubljana, Slovenia; 8 Faculty of Medicine, University of Ljubljana, Ljubljana, Slovenia

**Keywords:** Hereditary cancer syndromes, *BAP1*, Breast cancer, Immunotherapy

## Abstract

BAP1 cancer syndrome is a rare and highly penetrant hereditary cancer predisposition. Uveal melanoma, mesothelioma, renal cell carcinoma (RCC) and cutaneous melanoma are considered BAP1 cancer syndrome core cancers, whereas association with breast cancer has previously been suggested but not confirmed so far. In view of BAP1 immunomodulatory functions, *BAP1* alterations could prove useful as possible biomarkers of response to immunotherapy in patients with BAP1-associated cancers. We present a case of a patient with BAP1 cancer syndrome who developed a metastatic breast cancer with loss of BAP1 demonstrated on immunohistochemistry. She carried a germline *BAP1* likely pathogenic variant (c.898_899delAG p.(Arg300Glyfs*6)). In addition, tumor tissue sequencing identified a concurrent somatic variant in *BAP1* (partial deletion of exon 12) and a low tumor mutational burden. As her triple negative tumor was shown to be PD-L1 positive, the patient was treated with combination of atezolizumab and nab-paclitaxel. She had a complete and sustained response to immunotherapy even after discontinuation of nab-paclitaxel. This case strengthens the evidence for including breast cancer in the BAP1 cancer syndrome tumor spectrum with implications for future cancer prevention programs. It also indicates immune checkpoint inhibitors might prove to be an effective treatment for BAP1-deficient breast cancer.

## Introduction

BAP1 (BRCA1-associated protein) is an ubiquitin carboxy-terminal hydrolase, which functions both in the cellular nucleus and the cytoplasm [1]. It is involved in chromatin remodeling and DNA damage repair, particularly homologous recombination through its putative interaction with the BRCA1-BARD1 complex [2]. It also regulates cell cycle progression, programmed cell death, cell metabolism and immune response [3]. Loss of functional BAP1 can lead to tumorigenesis due to acquired genetic alterations or in the setting of BAP1 cancer syndrome (also referred to as the *BAP1*-tumor predisposition syndrome).

BAP1 cancer syndrome was first identified as a distinct cancer predisposition in families in which mesothelioma and uveal melanoma co-segregated with *BAP1* germline pathogenic variants [4]. Most of these are truncating variants with somatic inactivation of the second allele leading to BAP1 deficiency, evident as loss of immunohistochemical staining for BAP1 protein in tumor cell nuclei. BAP1 cancer syndrome is a rare but highly penetrant cancer predisposition syndrome, with 85% germline variant carriers developing at least one cancer by age 50 [5]. The penetrance is estimated to reach nearly 100% with increasing age [6]. Uveal melanoma is reported as the most common malignancy, seen in 28% of carriers, followed by mesothelioma, cutaneous melanoma and renal cell carcinoma (RCC) [7]. Basal cell carcinoma, cholangiocarcinoma, and meningioma are also considered likely BAP1 cancer syndrome-related cancers [6, 8]. 75% of patients develop typical benign skin lesions, i.e. melanocytic BAP1-mutated atypical intradermal tumors (MBAITs) or BAPomas, presenting as skin colored to reddish brown nodules [9]. There is some evidence for including breast cancer in the BAP1 cancer syndrome tumor spectrum, but its high prevalence in the general population is a confounder increasing the likelihood of chance association [10, 11].

Due to BAP1 immunomodulatory functions, as well as lymphocyte infiltration and inflammatory tumor environment seen in some BAP1-deficient tumors, *BAP1* alterations are seen as possible biomarkers that may help predict response to immunotherapy [11]. We present a case of a patient with BAP1 cancer syndrom*e* who developed early-onset, BAP1-deficient breast cancer and had a complete and ongoing response to monotherapy with a PD-L1 inhibitor.

## Methods

### Ethics declaration

Written informed consent was obtained from the patient for DNA analysis and publication of pseudoanonymised data contained in this report. Tumor tissue analysis was performed as part of the study approved by the Slovenian National Medical Ethics Committee (0120–280/2019/4, date of approval 14/06/2019).

### Next generation sequencing (NGS)

For germline genetic analysis, all exon regions and exon/intron boundaries ± 25 nucleotides of 94 cancer risk genes were enriched using Nextera DNA Library Preparation Kit in combination with the commercially available TruSight Cancer Panel (Illumina, San Diego, USA). The sample was sequenced using the Illumina MiSeqDx Sequencing System (Illumina). Read alignment and variant calling was conducted using MiSeq Reporter software. The tumor DNA sample was prepared using TruSight Tumor 170 (Illumina) assay and sequenced on the Illumina NextSeq 550 Sequencing System (Illumina). Read alignment and variant calling was performed using TruSight Tumor 170 Local App software v2 (Illumina). Variant annotation was performed with the use of Variant Studio software and Alamut Visual software (Interactive Biosoftware, Rouen, France). Tumor mutational burden (TMB) was defined as the number of somatic non-synonymous substitutions and small indels (insertions and deletions less than 20 nucleotides) in coding regions per megabase (mut/Mb).

### Immunohistochemistry

Immunohistochemical staining was performed on formalin-fixed, paraffin-embedded tumor tissue, using a mouse monoclonal antibody against the BAP1 antigen (C-4; Santa Cruz Biotechnology, Santa Cruz, CA), according to manufacturer’s instructions and standard operating procedures. Nuclear staining in stromal cells, ductal epithelial cells and lymphocytes served as internal control.

## Results

### Clinical presentation

**Fig. 1:**
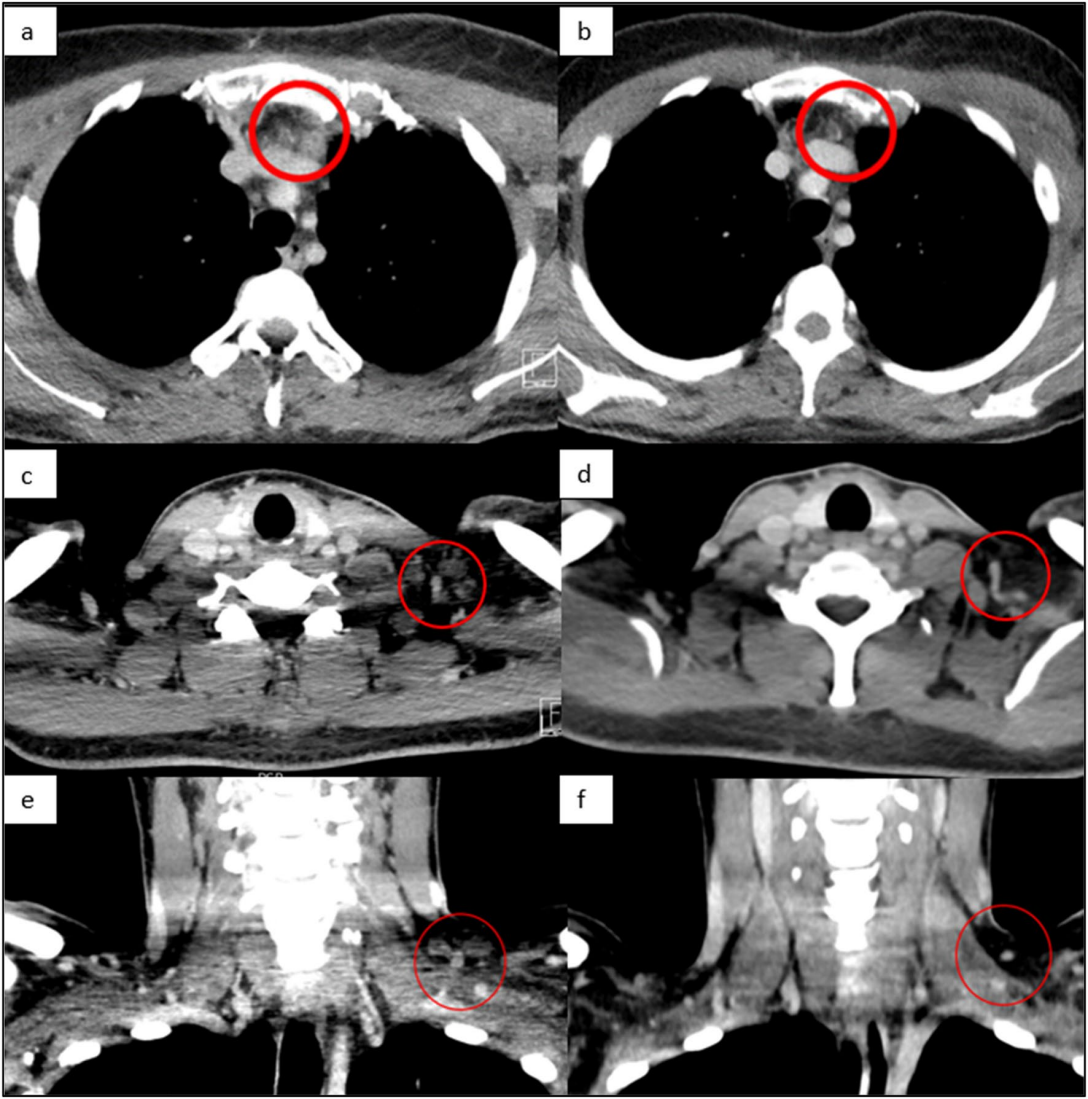
Response to treatment with immunotherapy. **a**, **c**, **e** CT images of the region anterior to the left brachiocephalic vein and the left supraclavicular region in axial and coronal cross sections showing metastatic lymph nodes prior to initiation of therapy with atezolizumab (initially in combination with nab-paclitaxel, which was later discontinued). **b** After 5 months of treatment, a complete response in the superior mediastinum was seen on CT. **d**, **f** After 8 months, there was also a complete response in the supraclavicular region

A 35-year-old female presented with a locally advanced cancer of her left breast. Core-needle biopsy demonstrated a triple negative invasive ductal breast carcinoma (TNBC) with a high proliferating factor Ki67 of 30%. Axillary lymph nodes (LNs) were also involved but no distant metastases were present at the time of diagnosis. She was treated with neo-adjuvant chemotherapy with anthracyclines and taxanes, and subsequently underwent a tumorectomy with left axillary dissection. Pathohistological examination of the excised specimen showed residual carcinoma with poor response to chemotherapy and tumor infiltration in 8 of 20 axillary LNs. Based on the large volume of residual disease she was treated with 8 cycles of pseudoadjuvant capecitabine. Twelve months after her initial diagnosis, while still undergoing treatment, a local recurrence was detected. CT scans showed metastases in the left supraclavicular region and infraclavicular LNs. Fine-needle aspiration biopsy of an infraclavicular LN confirmed a breast cancer metastasis. There was no response to treatment with radiotherapy. Based on the results of the IMpassion130 trial [12], which evaluated the role of atezolizumab in combination with chemotherapy in patients with TNBC and showed an important survival advantage for the PD-L1 positive subgroup, we tested the tumor tissue for PD-L1 expression. The tumor was found to be PD-L1 positive with 5–7% intratumoral lymphocytes expressing PD-L1, and combination therapy with atezolizumab and nab-paclitaxel was initiated in September 2019. One month later, a good partial response of affected LNs in the left supracvlavicular region was observed. CT scans, performed 5 and 8 months after treatment initiation revealed a complete response of lesions in the supra- and infraclavicular region (Fig. 1). Due to peripheral neuropathy and patient preference, nab-paclitaxel was discontinued in January 2020, but she continued on maintenance atezolizumab. She did not experience any immune-related adverse events. CT imaging in September 2020 showed no evidence of metastatic disease. At her check-up in January 2021 the patient was clinically still in remission.

### Genetic analysis and immunohistochemistry

**Fig. 2:**
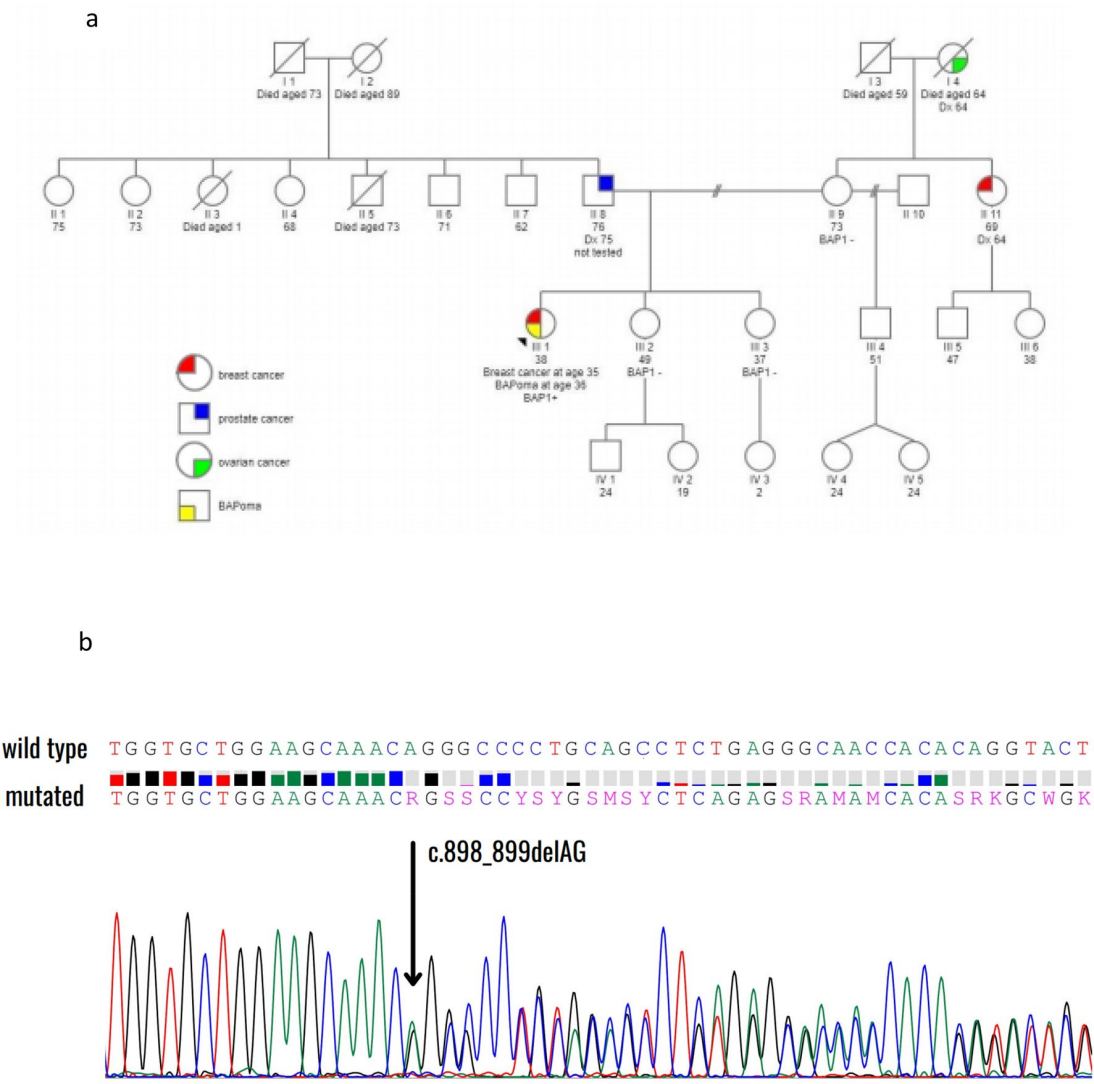
Family history and genetic testing. **a** Patient’s pedigree. The patient's two sisters and her mother do not carry the BAP1 PV. Her father has not been tested. There are no known cases of malignant mesothelioma, uveal or cutaneous melanoma or renal cell carcinoma in her family. **b** An electropherogram showing the heterozygous pathogenic variant c.898_899delAG in *BAP1*, detected in the patient’s blood sample

On germline genetic testing no pathogenic variants were detected in genes associated with hereditary breast and ovarian cancer (HBOC). Analysis of other cancer risk genes yielded a secondary finding —*BAP1*:c.898_899delAG p.(Arg300Glyfs*6) likely pathogenic variant (class 4) in heterozygous form. The patient was enrolled in a cancer surveillance program, in accordance with current recommendations for patients with BAP1 cancer syndrome [10]. On dermatological examination she was found to have several skin-colored nodules, one of which was surgically removed and confirmed to be a MBAIT. The patient’s two healthy sisters both tested negative for the *BAP1* variant. Her mother had a family history of breast and ovarian cancer but was herself healthy and tested negative for variants in HBOC genes as well as the *BAP1* variant. The patient’s father was unavailable for genetic testing. He subsequently developed a locally advanced prostate cancer. Our patient’s pedigree is shown in Fig. 2.

**Fig. 3:**
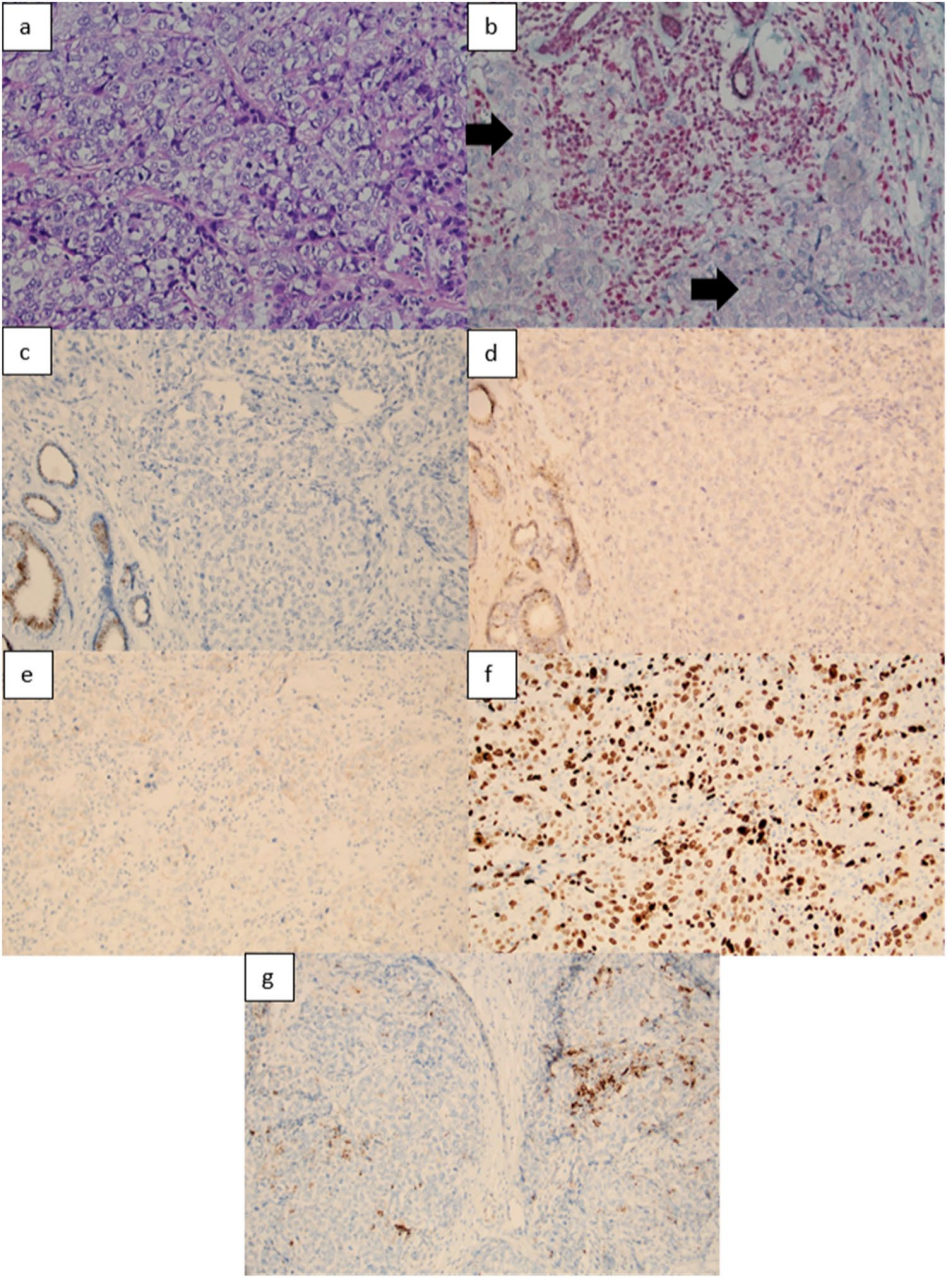
Tumor morphology and immunohistochemistry for BAP1, estrogen and progesterone receptors (ER, PR), human epidermal growth factor receptor 2 (HER2), Ki-67 antigen and programmed cell death ligand 1 (PD-L1). **a** Image shows a poorly differentiated breast carcinoma (hematoxylin and eosin, 100 × magnification). **b** Immunohistochemical staining for BAP1. BAP1 is lost in tumor cells (black arrows), and retained in ductal epithelial cells and lymphocytes that serve as internal controls. Additional immunohistochemical staining shows tumor cells are ER-negative (**c**) PR-negative (**d**) and HER2 negative—score 1 + (**e**). f Proliferation marker KI-67 is expressed by 30–40% of tumor cells. **g** Approximately 5–7% of intratumoral lymphocytes express PD-L1

Immunohistochemical staining performed on patient’s breast tumor tissue showed loss of BAP1 nuclear expression in tumor cells (Fig. 3). NGS analysis of DNA extracted from tumor tissue showed a low TMB (4 variants/Mb). A somatic *TP53* pathogenic variant c.332 T > G p.(Leu111Arg) was detected. Copy-number variation analysis indicated a partial deletion of exon 12 in *BAP1* could be the “second hit” causing inactivation of the wild-type *BAP1* allele.

## Discussion

We present a case of BAP1-deficient breast cancer arising in a patient with BAP1 cancer syndrome. The association of BAP1 cancer syndrome with breast cancer has proven difficult to demonstrate, with cohort studies yielding inconclusive results [6, 7, 10]. These studies were limited by small numbers of participants, which is understandable considering the rarity of BAP1 cancer syndrome. Breast cancer, occasionally with early onset has been described in several families, most notably in the study by Popova and colleagues, which was the first to report the association of *BAP1* germline pathogenic variants with RCC [8]. The *BAP1*-positive proband in one of their families developed bilateral early onset breast cancer, in addition to several RCCs. Her half-sister, who was also a carrier of the familial *BAP1* variant developed early-onset breast cancer as well. Interestingly, authors presented evidence for loss of heterozygosity at the *BAP1* locus in the proband’s breast tumor. However, the overall incidence of breast cancer in their *BAP1*-positive families was deemed insufficient to include it in the BAP1 cancer syndrome spectrum. *BAP1* germline variants are rare in patients with breast cancer undergoing genetic testing for hereditary cancer [10]. When testing for somatic variants, *BAP1* inactivation was seen in less than 1% of breast cancer cases, suggesting it is not a major breast cancer gene [10]. A study involving 760 unselected Australian breast cancer patients detected loss of immunohistochemical BAP1 expression indicating biallelic inactivation in three patients (0.4%) [13]. One of them had previously developed a breast cancer and another also had cutaneous melanoma, but patients were not screened for germline variants.

One of the intriguing aspects of BAP1-deficient tumors is their variable prognosis depending on tumor type. There is evidence that mesothelioma is less aggressive when it arises in the setting of BAP1 cancer syndrome, whereas the effect of somatic *BAP1* variants on mesothelioma prognosis is minimal [6]. BAP1-deficient uveal melanoma has a poor prognosis, particularly in cases due to somatic inactivation [6]. *BAP1* aberrations predispose to an aggressive type of RCC, with worse overall survival [14]. The analysis of transcriptome profiling data sets indicates that lower BAP1 expression imparts an unfavorable prognosis for breast cancer patients [15]. Some BAP1-associated tumors appear to have a poor prognosis due to their unresponsiveness to conventional therapy [16]. Therapeutic approaches targeting the role of BAP1 in cancer are currently under investigation, with on-going clinical studies assessing the effectiveness of PARP, EZH2 and HDAC inhibitors [7]. Immunotherapy with immune checkpoint inhibitors (ICI) elicits a response in 20–30% of unselected mesothelioma patients but only 5–15% of unselected patients with uveal melanoma [7]. Studies in peritoneal mesothelioma and RCC patients have shown BAP1-related tumors have an inflammatory microenvironment, with increased immune cell tumor infiltration and PD-L1 expression [11, 17]. Figueiredo et al. recently demonstrated BAP1-deficient uveal melanomas exhibit an immunosuppressive environment, with upregulation of regulatory T-cells leading to inhibition of cytotoxic T-cells and proposed anti-CD38/anti-CD74 compounds could be used in combination with ICI to increase the response rates to therapy [18].

Most breast cancers appear to be intrinsically resistant to treatment with immunotherapy with only a minority of patients responding to monotherapy with ICI [19]. Selecting patients most likely to respond is challenging. PD-L1 immunohistochemistry can produce inconsistent results, whereas TMB is relatively low in breast cancer. Additional biomarkers including variants in DNA damage repair genes are therefore being pursued [20]. As our patient’s TNBC expressed PD-L1, a combination treatment with ICI and a taxane was initiated. Even after discontinuation of nab-paclitaxel, she remained in remission with maintenance atezolizumab. To our knowledge, this is the first report of a patient with BAP1-deficient breast cancer responding to immunotherapy. We hypothesize that the tumor’s immunophenotype and the patient’s complete and durable response to ICI might be the result of BAP1-deficiency, making *BAP1* alterations a putative biomarker for response to immunotherapy in breast cancers that harbor them. A single case of a BAP1-defficient breast cancer responding to atezolizumab is by no means evidence of a role for BAP1 inactivation in response to immunotherapy, but we believe our findings are interesting and warrant further investigation.

Our report implies there is an association between BAP1 cancer syndrome and breast cancer. Additional epidemiological studies are needed to clarify the risk as this has important implications for patient management. Also, possible genotype–phenotype associations need to be explored. At the moment, breast cancer screening is offered to *BAP1* carriers on the basis of personal risk factors and family history. Even if the risk of developing breast cancer is low or moderate for most patients, screening might be advisable if BAP1-deficient breast cancers are confirmed to be associated with poor prognosis and/or warrant specific therapeutic approaches.

## Data Availability

Requests for data can be addressed to the corresponding author but need to be approved by the institutional Research Review Board.

## References

[1] Jensen D, Proctor M, Marquis S (1998). BAP1: a novel ubiquitin hydrolase which binds to the BRCA1 RING finger and enhances BRCA1-mediated cell growth suppression. Oncogene.

[2] Nishikawa H, Wu W, Koike A (2008). BRCA1-associated protein 1 interferes with BRCA1/BARD1 RING heterodimer activity. Can Res.

[3] Wang A, Papneja A, Hyrcza M (2016). Gene of the month: BAP1. J Clin Pathol.

[4] Testa J, Cheung M, Pei J (2011). Germline BAP1 mutations predispose to malignant mesothelioma. Nat Genet.

[5] Abdel-Rahman M, Rai K, Pilarski R (2016). Germline BAP1 mutations misreported as somatic based on tumor-only testing. Fam Cancer.

[6] Carbone M, Harbour J, Brugarolas J (2020). Biological mechanisms and clinical significance of BAP1 mutations in human cancer. Cancer Discov.

[7] Louie B, Kurzrock R (2020). BAP1: Not just a BRCA1-associated protein. Cancer Treat Rev.

[8] Popova T, Hebert L, Jacquemin V (2013). Germline BAP1 mutations predispose to renal cell carcinomas. Am J Human Genet.

[9] Haugh A, Njauw C, Bubley J (2017). Genotypic and phenotypic features of BAP1 cancer syndrome. JAMA Dermatol.

[10] Pilarski R, Carlo M, Cebulla C, Abdel-Rahman M (2021) BAP1 Tumor Predisposition Syndrome. In: Ncbi.nlm.nih.gov. https://www.ncbi.nlm.nih.gov/books/NBK390611/. Accessed 15 Apr 2021

[11] Shrestha R, Nabavi N, Lin Y (2019). BAP1 haploinsufficiency predicts a distinct immunogenic class of malignant peritoneal mesothelioma. Genome Med.

[12] Schmid P, Adams S, Rugo H (2018). Atezolizumab and nab-paclitaxel in advanced triple-negative breast cancer. N Engl J Med.

[13] Chui J, Singh A, Gill A (2017). Loss of BAP1 expression is very rare in breast carcinoma. Pathology.

[14] Ricketts C, Linehan W (2015). Gender specific mutation incidence and survival associations in clear cell renal cell carcinoma (CCRCC). PLoS ONE.

[15] Shahriyari L, Abdel-Rahman M, Cebulla C (2019). BAP1 expression is prognostic in breast and uveal melanoma but not colon cancer and is highly positively correlated with RBM15B and USP19. PLoS ONE.

[16] Oehl K, Vrugt B, Wagner U (2021). Alterations in BAP1 are associated with cisplatin resistance through inhibition of apoptosis in malignant pleural mesothelioma. Clin Cancer Res.

[17] Wang T, Lu R, Kapur P (2018). an empirical approach leveraging tumorgrafts to dissect the tumor microenvironment in renal cell carcinoma identifies missing link to prognostic inflammatory factors. Cancer Discov.

[18] Figueiredo C, Kalirai H, Sacco J (2020). Loss ofBAP1expression is associated with an immunosuppressive microenvironment in uveal melanoma, with implications for immunotherapy development. J Pathol.

[19] Nanda R, Chow L, Dees E (2016). Pembrolizumab in patients with advanced triple-negative breast cancer: phase Ib KEYNOTE-012 study. J Clin Oncol.

[20] Isaacs J, Anders C, McArthur H, Force J (2021). Biomarkers of immune checkpoint blockade response in triple-negative breast cancer. Curr Treat Options Oncol.

